# Organ-oriented proteogenomics functional atlas of three aquatic invertebrate sentinel species

**DOI:** 10.1038/s41597-023-02545-w

**Published:** 2023-09-21

**Authors:** Maxime Leprêtre, Davide Degli Esposti, Kevin Sugier, Anabelle Espeyte, Jean-Charles Gaillard, Nicolas Delorme, Aurélie Duflot, Isabelle Bonnard, Romain Coulaud, Céline Boulangé-Lecomte, Benoît Xuereb, Mélissa Palos Ladeiro, Alain Geffard, Olivier Geffard, Jean Armengaud, Arnaud Chaumot

**Affiliations:** 1 INRAE, UR RiverLy, Laboratoire d’écotoxicologie, 5 rue de la Doua, F-69625 Villeurbanne, France; 2 Université Paris-Saclay, CEA, INRAE, Département Médicaments et Technologies pour la Santé (DMTS), SPI, F-30200 Bagnols-sur-Cèze, France; 3 Université Le Havre Normandie, Normandie Univ, FR CNRS 3730 SCALE, UMR-I 02 SEBIO, Le Havre, F-76600 Le Havre, France; 4 Université de Reims Champagne-Ardenne (URCA), UMR-I 02 SEBIO, UFR Sciences Exactes et Naturelles, Campus Moulin de la Housse, BP 1039, 51687 Reims, France

**Keywords:** Proteomic analysis, Environmental impact

## Abstract

Proteogenomic methodologies have enabled the identification of protein sequences in wild species without annotated genomes, shedding light on molecular mechanisms affected by pollution. However, proteomic resources for sentinel species are limited, and organ-level investigations are necessary to expand our understanding of their molecular biology. This study presents proteomic resources obtained from proteogenomic analyses of key organs (hepatopancreas, gills, hemolymph) from three established aquatic sentinel invertebrate species of interest in ecotoxicological/ecological research and environmental monitoring: *Gammarus fossarum*, *Dreissena polymorpha*, and *Palaemon serratus*. Proteogenomic analyses identified thousands of proteins for each species, with over 90% of them being annotated to putative function. Functional analysis validated the relevance of the proteomic atlases by revealing similarities in functional annotation of catalogues of proteins across analogous organs in the three species, while deep contrasts between functional profiles are delimited across different organs in the same organism. These organ-level proteomic atlases are crucial for future research on these sentinel animals, aiding in the evaluation of aquatic environmental risks and providing a valuable resource for ecotoxicological studies.

## Background & Summary

The preservation of natural resources has become a crucial focus as our planet’s ecosystem faces significant challenges. In particular the increase of anthropogenic pollution, including toxic substances like heavy metals, nanoparticles, microplastics, pesticides, pharmaceutical drugs and other emerging contaminants poses a major threat to both human and ecosystem health^1^. The impact of the widespread exposure to these toxic substances on the health of living organisms, even at low doses, is largely unknown and difficult to manage from an environmental policy perspective. Their effects could even be exacerbated with global warming of our planet. Improving our understanding of the environment is more important than ever. The ‘One-Health’ concept aims to harmonize ecology, animal health, and human health knowledge and understanding of their inter-relationships for a better long-term quality of life^2^. In this context, the use of aquatic sentinel species viewed as scouts of water ecosystem health is gaining momentum to monitor the environmental quality with a great depth of integration, particularly to decipher and anticipate the impacts of chemical cocktail contamination on biodiversity.

Omics technologies such as genomics, transcriptomics, proteomics, and metabolomics offer a comprehensive molecular insight into the biological impact of environmental changes and pollution^3^. These technologies allow us to investigate changes in the genetic makeup, gene expression, protein levels, and metabolic pathways of sentinel species. The information gathered from these studies can enhance our understanding of the effects of environmental changes, including pollutions, on the health status of sentinel species. Development of molecular markers for precise biomonitoring is also an important outcome of such large scale molecular studies. It is also crucial to aid in developing conservation and management strategies to minimize their impact^3^. By means of combining data from RNA sequencing and shotgun mass spectrometry on proteins, proteogenomic methodologies have made possible to quickly identify protein sequences in a large variety of species without yet sequenced and annotated genomes^4^. In aquatic ecotoxicology, this approach can now be considered as standard for characterizing proteomes of sentinel species, as exemplified by pioneering studies such as the reproductive proteome of *Gammarus fossarum* and the immune proteome of *Dreissena polymorpha*^5,6^. Proteogenomics has also been used for deciphering the effects of contaminants on the proteomes of wild organisms. For example, these approaches have been employed to elucidate the basis of insecticide tolerance in the pollen beetle *Brassicogethes aeneus*^7^, as well as the effects of carbamazepine on the proteomes of the marine mussel *Mytilus galloprovincialis*^8^. However, proteomic resources for sentinel species remain limited, appealing to complement available information on biological functions impacted by pollutants or other environmental stressors that can be obtained through molecular investigations at the organ level.

The proteomic resources presented in this report correspond to proteogenomic analyses performed on relevant organs of three well-established aquatic sentinel species: *Gammarus fossarum* (Crustacean, Amphipod), *Dreissena polymorpha* (Mollusk, Bivalve), and *Palaemon serratus* (Crustacean, Decapod). These species are widely distributed in aquatic ecosystems (the two former are freshwater species and the latter is a marine species) and are found in most European countries^9,10^, and also in North America for the invasive bivalve. They have emerged as important ecotoxicological models for assessing the impact of pollution on freshwater wildlife and ecosystems. *P. serratus*, *G. fossarum*, and *D. polymorpha* are phylogenetically distant and come from the largest phyla of the animal kingdom (arthropods and mollusks)^11^. Using a combination of species-specific transcriptomes and shotgun proteomics, the proteomes of three organs (hepatopancreas/ hepatopancreatic caeca, hemolymph, and gills) from *P. serratus* and *G. fossarum*, as well as the gill proteome of *D. polymorpha*, were established and characterized. Overall, 3,891 and 2,419 proteins were experimentally validated from the three organs of *P. serratus* and *G. fossarum*, respectively, with a higher number of proteins identified in gills compared to digestive organs and hemolymph. In the case of *D. polymorpha*, proteogenomics identified 6,026 proteins in the gills. These comprehensive organ-level proteomic atlases were annotated in terms of putative function by sequence similarity, providing valuable insights into the biological pathways associated with each targeted organ. With over 90% of the proteins confidently annotated, the functional analysis revealed that analogous organs in the three species express partly functionally similar catalogues of proteins, while deep contrasts between functional profiles can be delimited across the different organs in the same organism. These observations highlight the value of such resources to capture biological processes at the molecular level in such sentinel non-model species. These proteomic atlases will serve as crucial references for future research on these sentinel animals and will aid in the assessment of aquatic environmental risks, contribute to a better understanding of molecular diversity in organisms, and provide a valuable resource for future ecotoxicological studies.

## Methods

### Study design

**Fig. 1:**
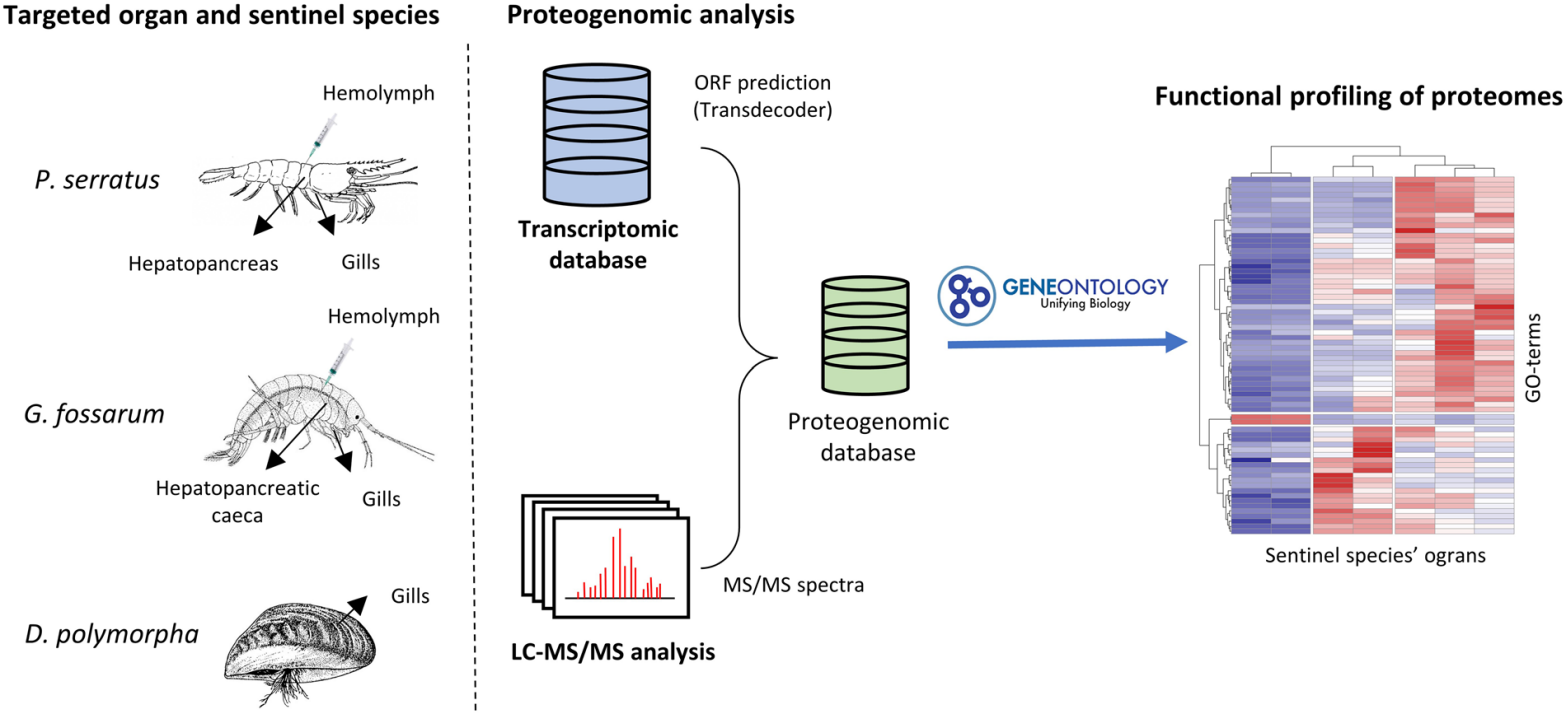
Proteogenomic workflow for the characterization and functional profiling of sentinel species organ proteomes.

The proteomes of three organs (hepatopancreas/hepatopancreatic caeca, hemolymph, and gills) from two crustacean species (*G. fossarum* and *P. serratus*) and the gill proteome from *D. polymorpha* were investigated using proteogenomics with a RNA-transcriptome informed database (Fig. 1). The study involved extracting proteins from tissue samples, tryptic digestion, and analyzing the resulting peptides using liquid chromatography-tandem mass spectrometry (LC-MS/MS) in Data Dependent Acquisition (DDA) mode. The DDA strategy was preferred over Data Independent Acquisition mode in order to have the highest possible confidence in the identification of peptides. MS/MS spectra were interpreted using open-reading frame databases derived from transcriptome databases specific to each species. To gain first insight into the functional roles of each organ-oriented proteomes for each species, the experimentally validated protein sequences were annotated with gene ontology (GO) terms weighted with normalized spectral abundance factor (NSAF) and compared through clustered heatmap analysis.

### Construction of a paired-end RNA-Seq library and illumina sequencing

The reference transcriptomes used *for G. fossarum* and *D. polymorpha* were generated as described in Cogne *et al*.^12^ and Leprêtre *et al*.^5^, respectively. For *P. serratus*, RNA Seq library construction and sequencing were performed by Genotoul (INRAE, France), using the NovaSeq sequencing instrument (Illumina). The library construction was performed from RNAs coming from three distinct tissues: hepatopancreas, cephalon and muscle. The TrueSeq Stranded (Illumina) protocol was used to construct the libraries, and RNA were sequenced using the paired-end reads 2 × 150 bp method on a SP flowcell lane. The reads were then assembled using the Trinity pipeline (version 2.3.0).

### Organ sampling from sentinel species for proteomic investigations

Adult specimens of *P. serratus* were collected by a fisherman (Prelev’Mar®), using specific traps, in the 2-nautical mile zone of Cherbourg (Normandy, France), in November 2020. The shrimps were transported to the laboratory within 3 h, in 30-liter plastic containers filled with natural seawater from the sampling site and kept oxygenated. Shrimps were kept some days into 80 L-aquariums, at density less than one specimen per liter, 33 of salinity, 18 ± 1 °C, with 16/8 h light/dark photoperiod, under constant oxygenation and filtration. During this period, shrimps were fed daily with a mix of krill and mysids (Europrix®). Male shrimps were kept in these containers until the organ sampling. A male specimen was anesthetized on ice. The hemolymph was collected from the pericardial sinus using a 1 mL syringe with a 1.5-inch, 23-gauge needle. Then, the hepatopancreas and gills were dissected. Samples were immediately snap-frozen in liquid nitrogen and stored at −80 °C.

For *G. fossarum*, adult specimens were collected in April 2021 from a bygone watercress farm in Saint-Maurice-de-Rémens (eastern central France) using 2- and 2.5-mm sieves. The organisms were stored in plastic bottles filled with ambient freshwater and immediately transferred to the laboratory. They were acclimated for two weeks in a 10-liter aquarium with a constant temperature of 12 ± 0.5 °C and a photoperiod of 8 h of darkness and 16 hours of light, with constant aeration. The organisms were fed with alder leaves (*Alnus glutinosa*) *ad libitum*. After two weeks, the hepatopancreatic caeca and gills from three male gammarids were dissected under an optical microscope. The hemolymph was sampled from the telson of gammarids using a modified microcapillary. The hemolymph from 25 organisms were pooled and transferred to 50 µl of Hanks’ Balanced Salt Solution (HBSS, Sigma). All samples were stored at −80 °C until proteomic investigation.

For *D. polymorpha*, zebra mussels between 20–25 mm long were collected from the lake of Der (Grand Est, France). The mussels were maintained in the laboratory for 24 h in 20-liter tanks filled with spring water Cristaline Aurèle (Jandun, France) with a constant temperature of 14 °C and controlled aeration. Gill tissues from individual mussel were dissected and stored at −80 °C for proteomic analysis.

For each organ of the different organisms the number of biological replicates used for MS/MS analyses are listed in the Table 1

**Table 1:** Summary of biological replicates, technical replicates, SDS-Page Fractions, and total MS/MS runs for each organ and species.

Biological samples		MS/MS analyses			
Sentinel species	Organs	Biological replicates	Analytical replicates	SDS-page fractions	Total MS/MS runs
*P. serratus*	Hepatopancreas	1	2	5	10
	Gills	1	2	5	10
	Hemolymph	1	2	5	10
*G. fossarum*	Hepatopancreatic caeca	3	1	1	3
	Gills	3	1	1	3
	Hemolymph	1 (25)	2	5	10
*D. polymorpha*	Gills	1	2	5	10

For the biological replicate of *G. fossarum* hemolymph, the number in brackets denotes the total number of hemolymph samples pooled.

### Protein extraction and in gel digestion

For each organ except *G. fossarum* gills and caeca, 20 µL of LDS sample buffer (Thermo) was added per mg of organ. The samples were first mechanically homogenized as previously described in Cogne *et al*.^13^ by bead-beating with one 3.2 mm steel bead per tube with a Precellys instrument (Bertin). Then, the steel bead was removed and replaced with a mixture of 0.1 mm silica beads and 0.5/0.1 mm glass beads as reported earlier^14^ for another cycle of bead-beating. Proteins were subjected to SDS-PAGE onto a 4–12% gradient 10-well NuPAGE gel (Invitrogen) for a 10 min migration at 200 V. The proteome slightly resolved along the molecular weight was subdivided into five fractions of equal volume of polyacrylamide band (Table 1). The proteins in each polyacrylamide band were reduced with dithiothreitol (DTT), alkylated with iodoacetamide, washed, and proteolyzed with Sequencing Grade Trypsin (Roche) in the presence of 0.01% ProteaseMAX surfactant (Promega) for 1 h at 50 °C. The proteolysis was stopped with the addition of trifluoroacetic acid (TFA) 5% to reach a final concentration of 0.5%.and subjected to trypsin proteolysis. For the *G. fossarum* gills and caeca samples, the same protein extraction protocol was applied, but the SDS-PAGE was performed for 5 min. Each proteome was treated as a single polyacrylamide band.

### NanoLC-MS/MS analysis

For all the samples except *G. fossarum* gills and hepatopancreatic caeca, the peptide samples extracted from one biological replicate and fractionated into five SDS-page bands were analysed in data-dependent acquisition mode with a Q-Exactive HF mass spectrometer (Thermo) coupled with an UltiMate 3000 LC system (Dionex-LC Packings). Peptides (200 ng) were desalted on-line and then resolved onto a nanoscale C18 PepMapTM 100 capillary column (LC Packings) with a 120-min gradient of CH3CN, 0.1% formic acid, at a flow rate of 0.2 µL/min. Peptides were analysed with scan cycles initiated by a full scan of peptide ions in the Orbitrap analyser, followed by high-energy collisional dissociation and MS/MS scans on the 20 most abundant precursor ions. Full scan mass spectra were acquired from m/z 350 to 1800 at a resolution of 60,000. Ion selection for MS/MS fragmentation and measurement was performed applying a dynamic exclusion window of 10 sec. Only 2+ and 3+ charged peptides were selected for fragmentation. Each peptide sample underwent duplicate analysis, with two technical replicates performed for each of the five SDS-page fractions (Table 1). So, ten nanoLC-MS/MS runs of 120 min were performed per organ to maximize the dataset size. For the *G. fossarum* gills and caeca samples, the peptides extracted from three biological replicate and fractionated into a unique SDS-page band were analysed with a single technical replicate using an Exploris 480 tandem mass spectrometer connected to a Vanquish Neo UHPLC (Thermo-Fisher) and operated in Data-Dependant Acquisition mode with parameters similar to those previously described^15^. The applied gradient was developed for 120 min (5%–25% of 0.1% HCOOH/99.9% CH3CN against 0.1% HCOOH/99.9% H2O), followed by a 5 min wash (25%–40%) and re-equilibration. Ions with 2+ or 3+ charges were selected for fragmentation applying a dynamic exclusion of 10 s.

### Proteogenomics database design and MS/MS interpretation

For all the samples except *G. fossarum* gills and caeca, proteomics data were interpreted against specific transcriptome databases following a two-step database strategy. A first round of search was performed to identify the most probable proteins and construct a more specific database by selecting proteins with at least one unique peptide. The second round of search was performed on this resulting database to confidently identify the proteins and obtain improved quantitation. The *P. serratus* initial database comprised 52,782 entries totalling 25,079,966 residues. The *P. serratus* final database comprised 7,203 entries totalling 4,585,752 residues. The *D. polymorpha* initial database was obtained after predicting with Transeq the most probably open reading frames from a transcriptomics database with 941,470 entries. A total of 246,689 protein sequences totalling 42,459,289 residues were selected. The *D. polymorpha* final database comprised 11,978 entries totalling 4,916,414 residues. The *G. fossarum* database was with 80,589 entries totalling 15,753,148 residues. For these searches, the mascot search engine was used with a mass tolerance of 5 ppm for parent ions and 0.02 Da for MS/MS fragment analysis, allowing for up to two missed cleavages and full-trypsin specificity. Modifications considered were carboxyamidomethylated cysteine (+57.0215) as static, and oxidized methionine (+15.9949) and deamidation of asparagine/glutamine (+0.9848) as dynamic modifications. Peptides with a p-value lower than 0.05 were validated. A protein was validated in the final search if two unique peptide sequences were detected in a specific organ. The false positive rate for protein identification was estimated to be less than 1% using a reverse decoy database and the same parameters.

### Estimation of protein abundances

For each proteome, protein abundances were estimated using the normalized spectral abundance factor (NSAF) method. This involved summing the spectral counts of protein-derived peptides obtained from peptide-to-spectrum assignments. NSAF values were calculated by dividing the spectral count of each protein by its molecular mass expressed in kDa, as previously described^16^. To express the NSAF values as a ratio, each protein’s NSAF was divided by the sum of all NSAF values in the organ proteome, resulting in a percentage of NSAF (%NSAF) for each protein which is a proxy of its molar abundance.

### Functional analysis of proteomes

Proteomic sequences validated by proteogenomics were annotated as follows. BLAST sequence similarity searches were carried out using the BLASTp module of the OmicsBox software (version 2.0) against the SWISSPROT database with no restrictions on taxonomy. From these results, gene ontology (GO) and GO-slims annotations were retrieved using the GO-mapping tool of OmicsBox. The validity of all functional annotations was ensured with an E-value threshold of 1 × 10^−3^. Finally, KEGG (Kyoto Encyclopedia of Genes and Genomes) pathways were predicted using the EggNOG-mapper tool, using default parameters as described by Huerta-Cepas *et al*. (2017).

To gain insight into functional differences between targeted organs, a comprehensive analysis of GO annotations was performed. For this purpose, functional GO-slims annotations were weighted with the %NSAF values calculated for each identified protein. To visually compare frequencies of GO-slim annotations between organs and species, a clustered heatmap was generated using the R package pheatmap.

## Data Records

### Transcriptomic databases

Transcriptomic data and ORF data for *G. fossarum* can be accessed from Cogne *et al*.^12^. Specifically, the transcriptome has been deposited in GenBank with the identifiers GHDA01000000^17^, while the ORF database referred as “T-GFBM” can be downloaded as FASTA files from figshare within the folder “YC02_Transcriptome translated ORFs”^18^. Read sequences were deposited in the European Nucleotide Archive under the accession number SRR808972^19^ for *P. serratus* and the NCBI Sequence Reads Archive under the accession number SRP448656^20^ for *D. polymorpha*. The open-access data repository Recherche Data Gouv^21^ provides reference transcriptomes for *P. serratus* and *D. polymorpha*, available as FASTA files named “*Palaemon*_*serratus*_transcriptome” and “*Dreissena*_*polymorpha*_transcriptome.” Additionally, the repository offers FASTA files of the translated coding sequence regions of contigs, which were generated by Transdecoder and used for MS/MS spectrum assignments in *P. serratus* and *D. polymorpha*. These files are named “*Palaemon*_*serratus*_ORF” and “*Dreissena*_*polymorpha*_ORF” respectively^21^.

### Proteomic data

All mass spectrometry data are readily accessible through the ProteomeXchange Consortium, available via the PRIDE partner repository^22–25^. The dataset identifiers corresponding to these data can be found in Table 2. Information regarding the number of biological replicates and technical replicates associated with the Pride repository can be found in Table 1. A synthetic Excel table (named “PRIDE_descriptions”) available from the data repository Recherche Data Gouv^21^ provides a comprehensive description of all the file names and their associated replica types in the PRIDE databases.

**Table 2:** Pride dataset identifiers of mass spectrometry data acquired from different organs of the three sentinel species.

Sentinel species	Organ	Dataset identifier	DOI accessions
*P. serratus*	Hepatopancreas	PXD031698^22^	10.6019/PXD031698
	Gills		
	Hemolymph		
*G. fossarum*	Hepatopancreatic caeca	PXD040344^23^	10.6019/PXD040344
	Gills		
	Hemolymph	PXD031777^24^	10.6019/PXD031777
*D. polymorpha*	Gills	PXD031681^25^	10.6019/PXD031681

For detailed insights into the identified proteins, their functional annotations, and estimated abundances based on NSAF values, Tables named “PS_proteins”, “GF_proteins”, and “DP_proteins” have been provided for organs from *P. serratus*, *G. fossarum*, and *D. polymorpha*, respectively. The excel files can be downloaded from data repository Recherche Data Gouv^21^ and include accession names of proteins linked to ORFs accessions, protein scores, percent peptide coverage of proteins, spectral counts of analytical replicates, NSAF values, Blast annotations, GO-terms and KEGG pathway assignments for each identified protein.

## Technical Validation

### Mass spectrometry quality control

Mass Spectrometry Quality Controls based on Pierce HeLa protein digest standard (Thermo) were incorporated at multiple points in the worklist and the performances of reproducibility were assessed in terms of peptide and protein identification. The correct calibration of the instrument was checked before starting the worklist and was verified by the average error done on the measures for each sample. The calibration error was in average below 0.5 ppm (0.44 ppm for *P. serratus* proteomes, 0.43 ppm for *D. polymorpha* proteomes, and 0.26 ppm for *G. fossarum* proteomes). The efficiency of proteolysis of samples, defined as less than 7% of 2 miss-cleavage, was tested for each sample.

**Fig. 2:**
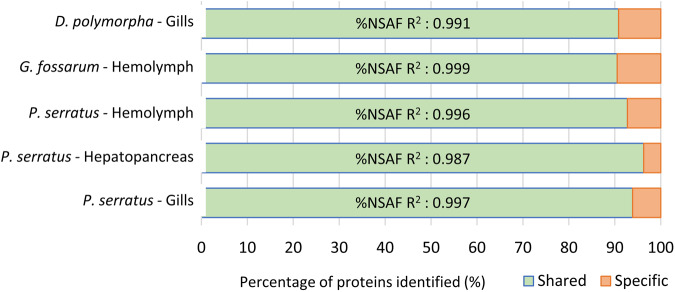
Percentage of validated proteins from species organs identified in both (shared) or single (specific) analytical replicates with unique peptides. Linear coefficient correlation (R^2^) between the %NSAF values of shared proteins, calculated in each analytical replicate, are also noted.

The reproducibility of analytical replicates was also evaluated by comparing the set of proteins identified in each replicate (Fig. 2). For each sample tested in duplicate, over 90% of the proteins validated from a specific organ (see section below) with a unique peptide sequence were identified in both replicates. Using linear regression correlations, the repeatability of the protein abundance estimations between replicate was also evaluated by comparing the %NSAF values of shared proteins calculated independently in each analytical replicate. Results showed that the lowest correlation coefficient (R^2^) between %NSAF values was observed for the replicates of *P. serratus* hepatopancreas samples (Fig. 2). However, even in this case, the R^2^ was greater than 98%, indicating a high level of reproducibility. Overall, these results showed that the protein identification and abundance estimation were highly reproducible between analytical replicates, which enhances the reliability and robustness of MS investigations.

### Peptide and protein validation

Decoy databases were generated to assess the false discovery rate (FDR) of identified peptides and proteins. A decoy database is a database of protein sequences generated by reversing or shuffling the target database of protein sequences. The decoy database contained the same number of proteins and similar properties as the target database but was composed of entirely random protein sequences. When searching for a shotgun experiment dataset against a decoy database, identified peptides that match to proteins in the decoy database represented false positives. In this study, an FDR of 1% was considered acceptable for peptide and protein identification in analyzed samples. In addition to controlling the FDR, the identification of at least two unique peptides (unambiguous in the whole database) was required for validating the identification of a protein in organs. Two unique peptides increase confidence in the identification of the protein since it is unlikely that two or more unrelated peptides will match to a single protein by chance. Moreover, identifying two or more unique peptides also allows for the estimation of the abundance of the protein, which can aid in understanding the biological function of the protein in the context of the sample being analyzed. In summary, the use of decoy databases and the identification of at least two unique peptides for validating protein identification in shotgun analyses ensured a high level of confidence in the identification of proteins in each organ of sentinel species.

**Fig. 3:**
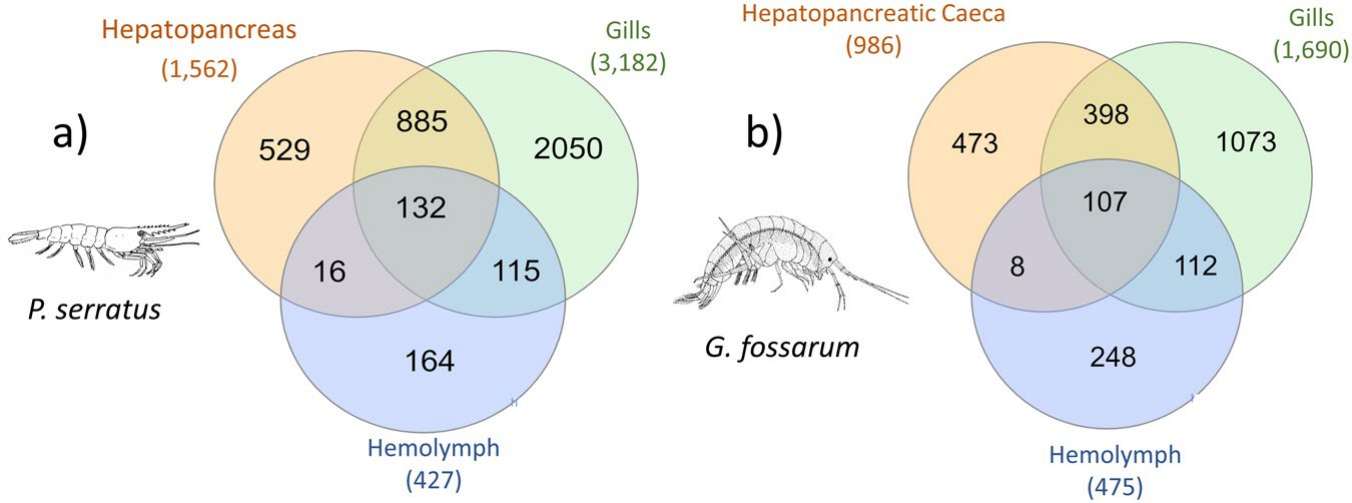
Venn diagram mapping shared or organ-specific proteins in *P. serratus* (**a**) *and G. fossarum* (**b**).

Using this confident strategy, a greater number of peptides and proteins were identified in the gills of *D. polymorpha* compared to the other samples investigated (Table 3). When comparing the proteomes of the three organs investigated in *P. serratus* and *G. fossarum*, a higher number of peptides and proteins were identified in the gill tissues compared to the digestive organs (hepatopancreas and hepatopancreatic caeca), followed by the hemolymphatic compartment with less than 500 proteins identified (Table 3). When mapping the proteins identified in different organs within a species, a small number of proteins were shown to be shared between organs, while the majority were unique to each organ. For example, in *P. serratus*, out of the 3,891 of proteins identified, only 132 were found to be common across all organs and more than 30% of the proteins were specific to each organ (Fig. 3a). The same trend was observed for the organ proteomes of *G. fossarum*, as shown in the Fig. 3b. These results underline that proteogenomics allowed to capture the proteome specificity of the various organs of species, proteomes that are logically tailored for performing the specific functions of each organ.

**Table 3:** Number of peptides – spectrum matches, peptides and proteins identified with FDR lower than 1% and at least 2 unique peptides in sentinel species organs.

Sentinel species	Targeted organs	Peptide-spectrum matches	Number of peptides	Number of validated proteins	Functional annotation (%)
*P. serratus*	Hepatopancreas	247,821	13,658	1,562	GO-terms: 96% KEGG: 64%
	Hemolymph	378,386	7,097	427	GO-terms: 87% KEGG: 49%
	Gills	467,457	34,260	3,182	GO-terms: 92% KEGG: 52%
*G. fossarum*	Hepatopancreatic caeca	57,457	7,863	986	GO-terms: 92% KEGG: 60%
	Hemolymph	226,661	4,781	475	GO-terms: 81% KEGG: 54%
	Gills	136,379	17,891	1,690	GO-terms: 92% KEGG: 61%
*D. polymorpha*	Gills	549,159	65,158	6,026	GO-terms: 86% KEGG: 47%

Percentage of proteins annotated to gene ontology (GO) annotations and KEGG pathways are also reported.

### Functional validity of organ proteomes

**Fig. 4:**
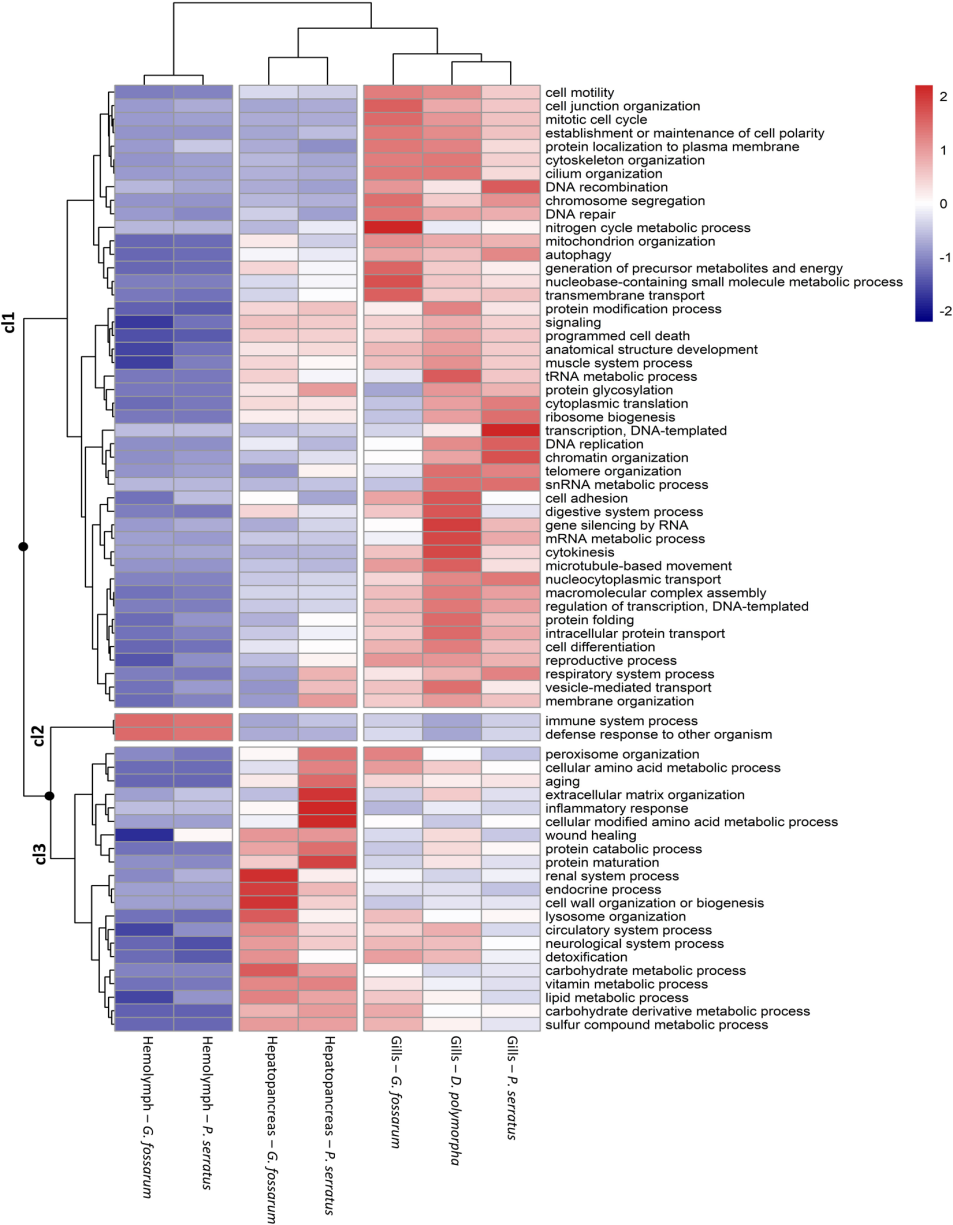
Clustered heatmap analysis performed on GO-slim annotations of the biological process class, weighted by the %NSAF calculated from each proteome. The rows were compared with the heatmap, and clustering was performed using the Euclidian method.

Functional analyses were performed to assess the functional significance of the proteomes established in the different target organs. For each proteome, functional profiling was conducted using ‘biological process’ related GO-slims annotated to about 90% of identified proteins with E-value cutoffs of 1.10^−3^ (Table 3). GO-slims were weighted with %NSAF values and compared across organ proteomes and species using a clustered heatmap analysis (Fig. 4). The heatmap showed that analogous organs in different species had similar functional profiles, grouping into three clusters of organs. The gills of all three species formed the first cluster, while the second cluster included the hepatopancreatic caeca and hepatopancreas from *G. fossarum* and *P. serratus*, and both hemolymph proteomes from *G. fossarum* and *P. serratus* were grouped together. The heatmap also revealed unique functional profiles specific to each organ type, distinguished by three functional annotation clusters. Overall, the functional profiles of proteomes are consistent with the expected biological functions of these organs, reinforcing the functional validity of the analyzed proteomic data.

The first functional annotation cluster (cl) showed that most of the GO-slims were found in higher abundance in the gill tissues of sentinel species compared to other organs (cl 1, Fig. 4). This cluster includes the GO-term ‘respiratory system process’, which is unsurprising given the crucial role that gills play in the respiration of aquatic organisms. Additionally, several GO-terms related to cellular transport mechanisms, such as transmembrane, intracellular, vesicle-mediated transport, and autophagy processes, were also identified. Cell transport mechanisms are vital for maintaining both osmoregulation and homeostasis in living organisms, as they enable cells to regulate the movement of molecules and ions in and out of cells, which is essential for maintaining stable internal conditions and proper physiological function^26,27^. Because gills are in direct contact with the external environment, they are known to play a critical role in osmoregulation and homeostasis in aquatic animals, regulating the exchange of gases, including oxygen and carbon dioxide, as well as the balance of ions and water^26,27^. The functional analysis also revealed that gill tissues exhibited a higher representation of GO-terms related to cell cycle mechanisms such as those involved in mitosis or DNA replication processes, suggesting that the gills have a high cell turnover rate compared to other organs^28,29^. Additionally, the over-representation of the GO-slim term “DNA repair” in gill tissues indicates that this organ may be subject to higher levels of genotoxic stress than other organs. As pinpointed by previous studies, this may be related to the direct exposure of gill tissues to environmental toxins and pollutants present in water^30,31^. A second cluster was characterized by a higher abundance of proteins annotated to GO-slims related to immune system processes in hemolymph compared to other organs (cl 2, Fig. 4). The hemolymphatic compartment, consisting mainly of immune cells (hemocytes) and plasma, plays multiple roles in the transport of immune effectors and the regulation of the immune response in invertebrate species^5^. Finally, the third group showed a higher abundance of GO-slims in the hepatopancreas and hepatopancreatic caeca of *P. serratus* and *G. fossarum*, respectively (cl 3, Fig. 4). As expected in these organs, these GO-slims were mainly related to metabolic processes, including the metabolism of amino acids, lipids, carbohydrates, and vitamins^32^. The GO-slim ‘detoxification’ was also overrepresented, indicating the role of these organs in removing harmful substances from the body also confirmed by the literature^33^.

## Usage Notes

Despite the significant findings obtained from the proteogenomic analyses conducted in this study, it is important to acknowledge certain limitations. One such limitation is the utilization of few biological and technical replicates for the MS/MS analysis of *P. serratus*, *G. fossarum* and *D. polymorpha* samples. This limitation arises due to practical constraints associated with the complex and resource-intensive nature of the experimental design. Future studies with larger sample sizes and multiple biological replicates would be valuable to validate and build upon the findings reported here. Such studies have the potential to provide a more comprehensive understanding of the proteomic landscape and further elucidate the variations that may occur within and between individuals of the same species.

Despite these limitations, it is important to emphasize that the present analyses represent an initial exploratory investigation that lays the groundwork for future studies. The findings of this study provide crucial insights into the proteomic profiles of the selected sentinel species and establish a strong foundation for future ecotoxicological investigations and environmental monitoring efforts.

## Data Availability

No custom code has been used to process the data. The search engine used to match MS/MS spectra to peptide sequences was Mascot Daemon 2.6.1 version (Matrix Science).
